# Couple-based expanded carrier screening provided by general practitioners to couples in the Dutch general population: psychological outcomes and reproductive intentions

**DOI:** 10.1038/s41436-021-01199-6

**Published:** 2021-06-10

**Authors:** Erwin Birnie, Juliette Schuurmans, Mirjam Plantinga, Kristin M. Abbott, Angela Fenwick, Anneke Lucassen, Marjolein Y. Berger, Irene M. van Langen, Adelita V. Ranchor

**Affiliations:** 1 Department of Genetics, University Medical Center Groningen, University of Groningen, Groningen, The Netherlands; 2 Clinical Ethics and Law, Faculty of Medicine, University of Southampton, Southampton, UK; 3 General Practice and Elderly Care Medicine, University Medical Center Groningen, University of Groningen, Groningen, The Netherlands; 4 Department of Health Psychology, University Medical Center Groningen, University of Groningen, Groningen, The Netherlands

## Abstract

**Purpose:**

The aim of expanded preconception carrier screening (ECS) is to inform any couple wishing to conceive about their chances of having children with severe autosomal or X-linked recessive conditions. Responsible implementation of ECS as reproductive genetic screening in routine care requires assessment of benefits and harms. We examined the psychological outcomes of couple-based ECS for 50 autosomal recessive (AR) conditions provided by general practitioners (GPs) to couples from the Dutch general population.

**Methods:**

Dutch GPs invited 4,295 women aged 18–40. We examined anxiety (State-Trait Anxiety Inventory, STAI-6), worry, decisional conflict (DCS) over time in participants declining GP counseling or attending GP counseling with/without testing.

**Results:**

One hundred ninety couples participated; 130 attended counseling, of whom 117 proceeded with testing. No carrier couples were identified. Before counseling, worry (median 6.0) and anxiety (mean 30–34) were low and lower than the population reference (36.4), although some individuals reported increased anxiety or worry. At follow-up, test acceptors reported less anxiety than test decliners (mean 29 vs. 35); differences in anxiety after testing compared to before counseling were not meaningful. Most participants (90%) were satisfied with their decision (not) to undergo testing.

**Conclusion:**

Some individuals reported temporarily clinically relevant distress. Overall, the psychological outcomes are acceptable and no barrier to population-wide implementation.

## INTRODUCTION

Advances in genomic technology enable relatively inexpensive and efficient carrier screening for multiple (rare) autosomal (AR) or X-linked conditions simultaneously, i.e., expanded carrier screening (ECS).^1^ ECS has the potential to enhance couples’ reproductive decisions by informing them about their risk of having children affected by severe genetic conditions and, when desired, to help carrier couples prevent conceiving or giving birth to an affected child.^2,3^ Couple members who carry the same AR condition have a 1 in 4 chance of having children affected by this condition in each pregnancy. Rather than offering carrier screening only to high risk groups based on ancestry or family history, ECS could be offered to any couple wishing to have children, i.e., a universal preconception test offer.^2^

The Department of Genetics, UMC Groningen, the Netherlands, developed an ECS preconception offer in 2013 targeting 50 severe early onset AR conditions without curative treatment.^4^ The selection criteria corresponded to the advice from an international expert meeting in Groningen in 2013 and professional recommendations.^2,4^ The test procedure was developed to identify carrier couples only, defined as both couple members being heterozygous for having a class IV (likely pathogenic) and/or class V (pathogenic) variant in the same gene conferring an autosomal recessive disorder. Couple-based ECS is advantageous: ECS is offered to couples from the general population without a known prior risk of being a carrier of an AR condition, and the chances of being a carrier for a particular condition in the test are generally low. Moreover, only couple-based results have utility for reproductive decision making, whereas individual carrier states do not. Furthermore, individual test results can invoke increased anxiety or worry, illness perceptions, and unnecessary resource use or physician workload.^5,6^ Additionally, couple-based ECS stimulates joint decision making and agreement on counseling and testing.^6^ The prior probability of being a carrier couple for a condition included in this test in the Dutch general population is approximately 1 in 150.^12^

As with any new screening test, it is important to weigh the potential harms and benefits before deciding to offer it to the eligible population.^2,7^ Potential harms include adverse psychological outcomes for those accepting and those declining this offer, and negative social implications such as routinization, overmedicalization of pregnancy, social pressures to take the test, and stigmatization or discrimination of carriers.^2,6,8^ The offer itself, the process of ECS, and the ECS result might result in feelings of psychological distress, such as worry and anxiety,^2,9–11^ and affect reproductive intentions and decisions. For example, receiving an ECS offer could confront eligible couples with new and unsolicited information about the risks of having a child with a rare severe genetic disorder. For those accepting the offer, the process of ECS and ECS results may cause psychological distress. Moreover, individuals and couples have to weigh up a range of factors before deciding to participate in or decline ECS, e.g., risk information, harms and benefits of undergoing the test, and reproductive options for identified carrier couples.^12^ Hence, decision making regarding participation in couple-based ECS is complex and could lead to feelings of decisional conflict or regret.^13,14^

We previously reported that provision of the UMCG test by motivated and trained general practitioners (GPs) was feasible, that most participants made an informed decision to proceed with ECS, and approximately 15% of couples accepted this offer.^15,16^ However, the psychological outcomes associated with an actual ECS offered to couples with a child wish from the general population are currently unknown. Yet, psychological outcomes are of great importance when judging whether future implementation of ECS would meet criteria for responsible implementation, e.g., the European Society of Human Genetics (ESHG) recommendation.^2^

To support decision making, we studied whether levels of anxiety and worry upon receiving the offer were within acceptable limits for acceptors and decliners of that offer. Secondly, we investigated the short-term and long-term effects on anxiety and worry in both test decliners and test acceptors. We also examined anticipated regret and participants’ satisfaction with the decision (not) to proceed with testing, decisional conflict, and the changes in reproductive plans of the test acceptors.

## MATERIALS AND METHODS

### ECS offer, sample, procedure

Between January and December 2016, 19 trained GPs invited 4,295 potentially eligible women aged 18–40 registered in their practices by letter to participate in ECS counseling and testing. Inclusion criteria were not being pregnant, having a (male) partner, and planning to have children with this partner. Interested couples had to make an appointment for pretest counseling about ECS (and general preconception care) with the inviting GP; both partners should attend the pretest counseling. After the counseling, couples could decide whether or not to proceed with testing. We used a couple-based ECS: both couple members provided blood samples and only the couple-based ECS result was disclosed, not the individual test results. The certified Genome Diagnostics Department, UMCG (ISO9001, UMCG hospital; ISO15189, Genome Diagnostics Department) performed the analysis of blood samples and communicated the results to the GPs. Couples with a known prior increased risk (e.g., suspect family history, consanguinity) were referred to Department of Genetics, UMCG, for counseling and possibly testing. If a carrier couple was found, GPs should discuss referral for post-test counseling, as this is a formal indication to be offered counseling at a clinical genetics center. All participating couples could contact a genetic counselor if they requested additional information, pretest or post-test.

### Design, subgroups

**Fig. 1:**
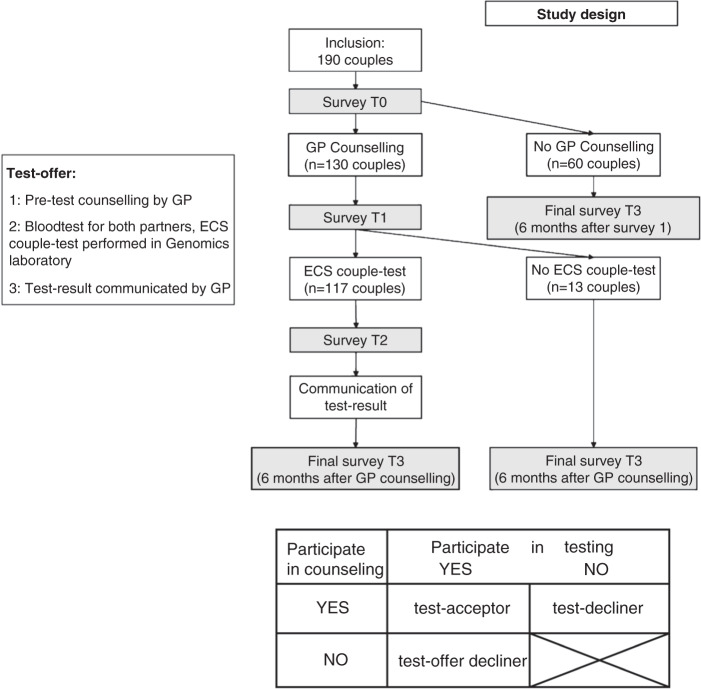
Overview of study design and offer. Offer (box), study design (longitudinal study), and number of couples through the study flow. The measurements points are T0, T1, T2, and T3. Three subgroups can be distinguished: test offer decliners (participants who did not attend GP counseling and therefore did not undergo expanded carrier screening [ECS]; 60 couples), test decliners (participants who attended GP counseling, i.e., initially accepted the offer, but declined ECS after GP counseling; 13 couples), and test acceptors (individuals who attended GP counseling and proceeded with testing; 117 couples).

Figure 1 depicts the longitudinal study design with four measurements: after receiving the offer (T0), after pretest counseling (T1), after testing (T2), and at 6 months after GP counseling or survey T0 (T3). Three subgroups were distinguished. *Test offer decliners* were participants who did not attend GP counseling and therefore did not undergo ECS but were willing to fill out the survey; they received two online surveys (T0, T3). *Test decliners* were participants who accepted the offer and attended GP counseling, but declined ECS after counseling; they received three surveys (T0, T1, T3). The *test acceptors* were the individuals who attended GP counseling and proceeded with testing; they received all four surveys. Further study details have been reported elsewhere.^15,16^

### Psychological outcomes, measures, instruments

Supplementary Table S1 details which concepts were measured over time. We asked participants to fill in the surveys independently from their partners using Roqua for confidential data collection.^17^ Appendix S1 details the concepts measured, instruments, and scoring models. Supplementary Table S2 lists the questions on anxiety, worry, and reproductive intentions.

Anxiety was measured using the 6-item short form State-Trait Anxiety Inventory (STAI-6) and transferred to prorated 20-item STAI scores (score range 20–80).^18^

Worry regarding being a carrier couple was measured using an adaptation of the 6-item Cancer Worry Scale;^19^ the score range is 6–24. Decisional conflict regarding test participation was measured using the 16-item Decisional Conflict Scale (DCS).^20,21^

Anticipated decisional regret (T0) was measured with one item: “I think that I would regret not having taken part in this offer later on.” Being satisfied with one’s decision (T3) whether to undergo couple-based ECS was measured using the DCS-item “I am satisfied with my decision." Factors that potentially influenced psychological outcomes were sociodemographic, relationship, health-related variables, and perceived control over life (7-item Pearlin Mastery Scale [PMS]^22^). Reproductive intentions were measured according to Lakeman et al.^10^

### Analysis

Psychological and other variables at any time point were summarized using the mean (SD) for normally distributed variables, median (IQR) for variables with skewed distributions, and *n* (%) for nominal/ordinal variables. To study whether anxiety and worry scores were within acceptable limits, mean anxiety at T0 between the joint group of test offer acceptors and test offer decliners was compared with the independent Student’s *t*-test. Mean anxiety was compared with the population reference score of 36.4 (35.4–37.3)^23^ using the independent Student’s *t*-test. Clinically relevant STAI scores were defined as the proportion of STAI scores ≥40. Worry scores between the joint group of test offer acceptors and test offer decliners at T0 were compared with the Mann–Whitney *U*-test. The long-term effects in anxiety and worry were estimated as the within-person change in anxiety and worry scores between T0 and T3 and compared within groups with the paired Student’s *t*-test and the Wilcoxon signed rank test, respectively. Short-term effects in anxiety and transformed worry scores between the groups over time were analyzed with repeated measurements analysis (linear mixed modeling, covariance structure: unstructured). The magnitude of short-term effects was derived from the beta-coefficients of the time-related variables.

Mean (SD) DCS scores were interpreted using the reference values (Appendix S1). Effect sizes were interpreted using Cohen’s *d*: small (0.2) medium (0.5), and large (0.8).^24^ A *p* value <0.05 (two-sided) was considered a statistically significant difference.

## RESULTS

### Participants

A total of 191 couples gave written consent. One couple was excluded before GP counseling due to unexpected pregnancy. Thus, we included 190 couples/380 participants. Response rates were 358/380 (93%) for T0, 238/260 (92%) for T1, 193/234 (82%), for T2 and 227/358 (64%) for T3. As Table 1 shows, test offer decliners were more likely to already have children and less likely to accept the ECS offer (intention) than the individuals who attended pretest counseling. The test decliners more often were planning a pregnancy within 6 months, were less often married or in a civil partnership and more frequently lived together, and less frequently suffered from a chronic condition compared to the test offer decliners and test acceptors. The test acceptors were on average higher educated and reported excellent health more frequently compared to the other groups.

**Table 1:** Sociodemographic, reproductive, and health characteristics by group^a^.

Characteristics	All *N* = 380	Test offer decliners *N* = 120	Test decliners *N* = 26	Test acceptors *N* = 234
Age (years), mean (SD)	29.1 (5.5)	28.7 (5.4)	30.1 (5.2)	29.3 (5.5)
Sex *n* (%)				
Female	185 (52.0)	55 (55.6)	13 (52.0)	117 (50.0)
Male	173 (48.0)	44 (44.4)	12 (48.0)	117 (50.0)
Age category *n* (%)				
18–24 years	69 (19.3)	23 (23.2)	3 (12.0)	43 (18.4)
24–32 years	180 (50.3)	46 (46.5)	11 (44.0)	123 (52.6)
>33 years	109 (30.4)	30 (30.3)	11 (44.0)	68 (29.1)
Religiosity *n* (%)				
Yes	84 (23.5)	19 (17.4)	7 (28.0)	58 (24.8)
Educational level *n* (%)				
Basic	25 (7.0)	11 (11.1)	0 (0.0)	14 (6.0)
Intermediate	178 (49.7)	61 (61.6)	18 (72.0)	99 (42.3)
High	155 (43.3)	27 (27.3)	7 (28.0)	121 (51.7)
Marital status *n* (%)				
Married/civil partnership	77 (21.5)	18 (31.3)	0 (0)	59 (25.2)
Living together	196 (54.7)	50 (50.5)	20 (80.0)	126 (53.8)
Not living together	90 (25.1)	31 (18.2)	5 (20.0)	49 (20.9)
Children *n* (%)				
Yes	55 (15.4)	24 (24.2)	3 (12.0)	28 (12.0)
Relationship satisfaction^b^				
Median (IQR)	9 (8–9)	8 (8–9)	9 (8–10)	9 (8–9)
Timing of next pregnancy *n* (%)				
<0.5 years	56 (15.6)	21 (21.2)	10 (40.0)	25 (10.7)
0.5–2 years	103 (28.8)	39 (39.4)	2 (8.0)	72 (30.8)
2–5 years	126 (35.2)	24 (24.2)	7 (28.0)	95 (40.6)
≥5 years	36 (10.1)	9 (9.1)	5 (20.0)	22 (9.4)
Unsure	27 (7.5)	6 (6.1)	1 (4.0)	20 (8.5)
Self-rated health *n* (%)				
Excellent	90 (25.1)	17 (17.2)	3 (12.0)	70 (29.9)
Very good	129 (36.0)	34 (34.2)	11 (44.0)	84 (35.9)
Good	127 (35.5)	44 (44.4)	10 (40.0)	73 (31.2)
Moderate	12 (3.4)	4 (4.0)	1 (4.0)	7 (3.0)
Poor	0 (0.0)	0 (0.0)	0 (0.0)	0 (0.0)
Do you suffer from a chronic condition? *n* (%) No	218 (60.9)	56 (56.6)	18 (72.0)	144 (61.5)
Any experiences with hereditary conditions in your family/friends? *n* (%)				
No experience	252 (70.4)	73 (73.7)	16 (64.0)	163 (69.7)
Did you have genetic testing and counseling in the past? *n* (%)				
Yes	13 (3.6)	2 (2.0)	4 (16.0)	7 (3.0)
Perceived control (PMS), mean (SD)	28.1 (3.9)	27.5 (3.8)	26.5 (3.7)	28.4 (3.9)
Intention to take part in the offer^c^ *n* (%)				
Likely	306 (86.2)	67 (69.1)	22 (91.7)	217 (92.7)
Neutral	30 (10.7)	15 (15.5)	1 (4.2)	14 (6.0)
Unlikely	19 (5.4)	15 (15.5)	1 (4.2)	3 (1.3)

*IQR* interquartile range, *SD* standard deviation.

^a^Missing data: test offer decliners: *n* = 22; test decliners: *n* = 1.

^b^A higher score represents higher relationship satisfaction.

^c^Missing data: test offer decliners: *n* = 23, test decliners: *n* = 2.

### Psychological outcomes after receiving the offer (T0)

#### Anxiety

**Fig. 2:**
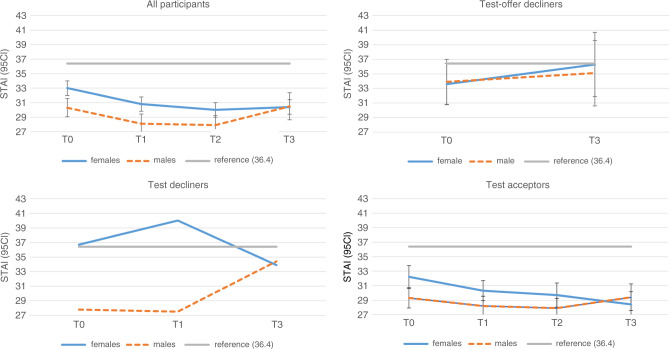
Mean State-Trait Anxiety Inventory (STAI-6) scores (with 95% CI of mean) by group over time. Mean levels of anxiety (prorated STAI scores) over time (T0, T1, T2, T3) for the total group (all participants), and the three subgroups: test offer decliners, test decliners, and test acceptors. For each group, the course of anxiety is distinguished between female (solid line, blue) and male (dashed line, orange) respondents. The error bars represent the limits of the 95% confidence interval of the mean. The population reference value of 36.4 is based on De Jong-Potjer et al.^23^ Summary data of the between-group differences at T0 and T3 can be found in Supplementary Tables S3 and S4. Summary data of the within-group differences between T3 and T0 can be found in Supplementary Table S5.

Figure 2 shows the mean anxiety scores per group over time (Supplementary Table S3). At T0, the mean STAI scores were significantly lower than the population reference of 36.4 for the test offer decliners (*p* = 0.02) and test acceptors (*p* < 0.001), and comparable to the reference for the test decliners (*p* = 0.09).

The test offer decliners reported higher mean anxiety levels than the individuals who accepted the offer (mean difference 2.76, *p* = 0.010; effect size 0.32). Individuals who did not proceed with testing (i.e., test offer decliners and test decliners) reported higher mean anxiety at T0 than the test acceptors (mean difference 2.73, *p* = 0.007; effect size 0.19). Moreover, at T0, the test offer decliners reported more frequently relevant anxiety (STAI score ≥40) compared to the joint group of test decliners and test acceptors (*p* = 0.017). The joint group of test offer decliners and test decliners reported more frequently relevant anxiety compared to the test acceptors (*p* = 0.008).

#### Worry

**Fig. 3:**
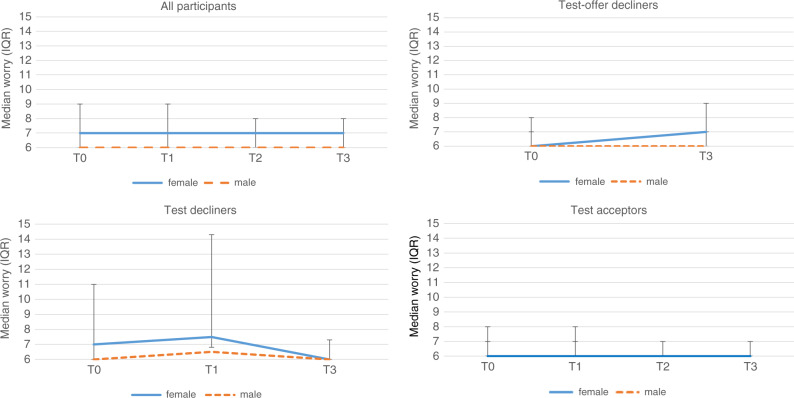
Median worry scores (adapted 6-item Cancer Worry Scale (CWS) scores) by group over time. Median levels of worry (adapted CWS scores) over time (T0, T1, T2, T3) for the total group (all participants), and the three subgroups: test offer decliners, test decliners, and test acceptors. For each group, the course of worry is distinguished between female (solid line, blue) and male (dashed line, orange) respondents. For the total group (all participants) and the test acceptors, note that the median worry scores for female and male respondents overlap. The error bars represent the limits of the interquartile range (1st and 3rd quartile). Summary data of the between-group differences at T0 and T3 can be found in Supplementary Tables S3 and S4. Summary data of the within-group differences between T3 and T0 can be found in Supplementary Table S5.

Figure 3 shows the median (IQR) worry scores for each group over time (Supplementary Table S3). At T0, the worry scores were not significantly different between the test offer decliners and the joint group of test decliners and test acceptors (*p* > 0.99; effect size 0.03), nor between the joint group that did not proceed with testing and the test acceptors (*p* = 0.80, effect size 0.35).

### Psychological outcomes: long-term differences (T3–T0)

#### Anxiety

At T3, the mean STAI scores were comparable to the population reference of 36.4 for the test offer decliners (*p* = 0.77) and the test decliners (*p* = 0.51) but significantly lower for the test acceptors (*p* < 0.001) (see Fig. 2, Supplementary Table S4). At T3, the test offer decliners reported higher mean anxiety levels than the test acceptors (mean difference 7.08, *p* < 0.001; effect size 0.79). The same trend was seen for the joint group that did not proceed with testing. They reported higher mean anxiety than the test acceptors (mean difference 6.77, *p* < 0.001, effect size 0.74). Clinically relevant anxiety was reported more frequently among the test offer decliners than the test acceptors (37.8% vs. 12.7%, *p* < 0.001) and among the joint group of test offer decliners and test decliners compared to the test acceptors (35.2% vs. 12.7%, *p* < 0.001). The within-group differences and effect sizes in STAI scores between T0 and T3 were small (Supplementary Table S5), suggesting that T3 and T0 were comparable.

#### Worry

As Fig. 3 (Supplementary Table S4) shows, the median worry scores of test offer decliners and test decliners at T3 were comparable to those of the test acceptors (median 6; *p* = 0.59 and *p* = 0.61, respectively); effect sizes were small. Moreover, the worry scores of the joint group who did not proceed with testing and the test acceptors were also comparable and effect sizes were small.

For all three groups, the within-group differences in worry scores were not significantly different between T0 and T3 (test offer decliners: *p* = 0.33, test decliners: *p* = 0.14; test acceptors: *p* = 0.90) (Supplementary Table S5). The effect sizes were all <0.20, suggesting that long-term differences in worry within groups are negligible.

### Psychological outcomes: short-term differences between T0 and T3

#### Anxiety

After adjusting for covariables, test offer decliners and test decliners reported higher mean STAI scores compared to the test acceptors (beta 4.23, *p* = 0.005; and beta 3.52, *p* = 0.22, respectively; Supplementary Table S6). Moreover, individuals who reported higher anxiety at T0 were more likely to report higher anxiety at T3 (beta 0.49, *p* < 0.001). After adjustment, there were no significant differences in mean STAI scores over time, suggesting that short-term differences in anxiety were absent.

#### Worry

After adjustment for covariables, test decliners had significantly lower worry than test acceptors (beta −0.52, *p* = 0.003) (Supplementary Table S6). Individuals who reported higher worry at T0 were more likely to report higher worry at T3 (beta 0.15, *p* = 0.001). Adjusted results showed no significant differences in mean worry scores over time, except for the test decliners: those who did not proceed with testing after counseling were more worried at T1 than those who proceeded with testing (beta 0.63, *p* = 0.003). This suggests the presence of a short-term increase of worry at T1 for the group of test decliners.

### Decision making

#### Decisional conflict

**Fig. 4:**
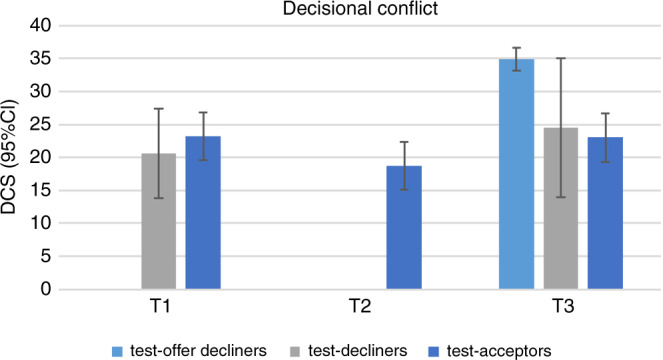
Mean Decisional Conflict Scale scores (with 95% CI of mean) by group over time. Mean levels of Decisional Conflict Scores (DCS) over time (T1, T2, T3) for the three subgroups: test offer decliners, test decliners and test acceptors. The error bars represent the limits of the 95% confidence interval of the mean. Note that DCS was not measured for all groups at all measurement points (Supplementary Table S1 and Fig. 1). Summary data can be found in Supplementary Table S7.

After counseling (T1) most individuals rated decisional conflict as low to moderate (Fig. 4). None of the test decliners and 7.5% of the test acceptors rated decisional conflict as high (Supplementary Table S5). At T3, high decisional conflict was reported more frequently in test offer decliners than in test acceptors (38.6% vs. 8.8%, *p* < 0.001). Decisional conflict rates were not significantly different between the test offer decliners and test acceptors (*p* = 0.67). Moreover, most test decliners and test acceptors indicated low/moderate levels of decisional conflict (about 90%), whereas about 80% of test offer decliners reported moderate/high levels of decisional conflict. The correlation coefficients between the DCS score and the STAI-6 score were 0.19–0.36, and between the DCS score and the worry score 0.14–0.19.

#### Anticipated regret, satisfaction

At T0, test offer decliners reported anticipated regret regarding test participation less frequently than the test decliners (35.5% vs. 58.3%, *p* = 0.04) and test acceptors (35.5% vs. 55.0%, *p* < 0.001) (Supplementary Table S7). At T3, 222 (97.8%) individuals were satisfied with their decision irrespective of what that decision was. Four test offer decliners were dissatisfied with their decision not to undergo testing, of whom two had anticipated regret at T0. One test acceptor with anticipated regret at T0 was dissatisfied with the decision to have ECS.

#### Reproductive intentions

No carrier couples were found. For 150/168 (89.3%) of the test acceptors, the ECS result had not affected their reproductive plans. Four individuals (4.2%) were not sure about whether the test result had changed their reproductive plans. The remaining 14 individuals (8.4%) indicated that they were more certain about having children with this partner.

## DISCUSSION

We investigated the psychological outcomes of a couple-based ECS offer to couples from the general population who wish to conceive. After receiving the offer, all study participants reported mean anxiety scores lower than or comparable to the population reference. Mean worry was stable and low over time. Long-term differences in anxiety and worry were absent. Short-term increased worry was only seen after GP counseling (T1), in the group that received counseling but declined testing. Moreover, individuals who attended GP counseling and proceeded with testing reported less anxiety and worry compared to those who did not undergo testing. Hence, at the group level, the psychological impact of offering ECS to the eligible population is acceptable. However, at the individual level, a minority of couple members reported clinically relevant increased anxiety, especially among the test offer decliners and test decliners. Decisional conflict was low/moderate for most test acceptors and test decliners, but moderate/high at follow-up for most test offer decliners. Overall, meaningful adverse psychological outcomes are absent for the group as a whole and its subgroups. This does not imply that our test offer was fully anxiety-free or worry-free. Rather, it suggests that the test offer did not reach worrisome or relevant levels. Reasons for and against accepting the test offer have been reported elsewhere^15^ and show substantial heterogeneity.

### Anxiety and worry

Despite differences in setting, study design, and included couples, our results on anxiety are comparable with the study of Kraft et al. that showed that anxiety scores at T0 were low and remained stable and low in women who participated in preconception ECS and received negative (normal) results.^25^ Furthermore, the test offer decliners and test decliners in our study on average reported higher anxiety scores than the test acceptors. This is also comparable to the study of Kraft et al., who found higher anxiety levels in the group that did not undergo ECS but just received regular care compared to those undergoing ECS.^25^ Metcalfe et al. studied preconception and prenatal fragile X carrier screening, and reported higher baseline (36.3–38.7) and higher 1 month follow-up anxiety scores (34.3–34.5) in nonpregnant women compared to our study and Kraft et al. These scores were still higher when adjusted for the 3.3% non-normal ECS results.^13^ Differences in anxiety could relate to the included population (women 18–70 years vs. 18–40 years in our study, women only vs. couples, individual vs. couple-based ECS result, large city/urban vs. large city/countryside), differences in information and counseling materials, or the conditions screened for (fragile X vs. 50 AR conditions). About 19% of individuals reported clinically increased anxiety, which is comparable to the 17.2% (negative results) and 22.3% (untested individuals) at 3–6 months follow-up as reported by Honnor et al. in a study about cystic fibrosis (CF) carrier screening in the general population.^26^ However, it is unclear what their definition of “raised anxiety” is. These proportions are comparable to or slightly higher than the 15–19% (baseline) and 12–16% (1 month follow-up) reported by Metcalfe et al. in nonpregnant women using the Depression Anxiety Stress Scale.^13^

The proportion of individuals who did not proceed with testing and reported clinically relevant anxiety (about 10–40%) was larger than that in test acceptors (9–20%). Metcalfe et al. found the same trend: clinically relevant anxiety is more frequently reported in individuals who decided not to participate in testing.^13^ One explanation is that these relatively high percentages may have been caused by small numbers and selective response. The finding that test offer acceptors show lower levels of anxiety compared to test offer decliners might indicate that especially people with low(er) anxiety levels have accepted the offer. Additionally, baseline anxiety could present a psychological barrier to enter ECS. Elevated anxiety at baseline could simply indicate that a certain proportion of people in the general population are more anxious than others (personality trait). It could also relate to the selection process or information policy that preceded counseling. In that case, tailored information and/or a decision tool might help to lower anxiety.

Worry was not reported in the Honnor et al. and Metcalfe et al. studies. Kraft et al. studied worry about test accuracy, concern for family members, and privacy concerns about the test result. Despite differences in concepts and instruments used, their mean worry and concerns scores are low and stable over time, a finding comparable to our study.^4^

### Decisional conflict, satisfaction, regret

After counseling and at follow-up, most test decliners and test acceptors hardly felt conflicted about their decision to undergo testing. In contrast, almost 40% of test offer decliners reported high decisional conflict at follow-up. This is in line with Metcalfe et al.: high decisional conflict and decisional uncertainty occur more frequently among those not tested.^13^ Surprisingly, decisional satisfaction was high irrespective of the decision taken. Despite small numbers, it is suggestive that 4 of 5 dissatisfied individuals were test offer decliners. Possible explanations for the high decisional conflict rates among test offer decliners include the following. First, the test offer decliners possibly felt unsure about whether to undergo testing. O’Connor et al. demonstrated that higher DCS scores are found in people who are unsure about participation in screening or delay their decision.^27^ This could also apply to our test decliners, as most of them had a positive intention towards accepting this offer at baseline but room for delay was minimal as couples had to make a GP appointment within one month. Hence, higher DCS scores may be a study artifact, at least partly caused by the temporary ECS offer, that may be absent or lower in a repeat offer after nationwide implementation. Secondly, the test offer decliners, while being approached with the 6-month survey, may have been confronted once more with a difficult to make decision, or an issue they considered already dealt with in the past. DCS showed low correlations with anxiety and worry.

We did not investigate if the written and online information included with the offer was sufficient to facilitate decision making. We previously showed that informed choice was reached (>90%),^16^ which suggests that our information strategy indeed facilitated decision making.

### Reproductive intentions

Our results regarding reproductive intentions agree with other studies: over 90% of test acceptors identified as noncarrier couples did not intend to change their reproductive plans and some felt more certain.^10,25^ This suggests a reassuring impact on reproductive decisions for those with a negative ECS. However, this may lead to false reassurance if couples do not understand the residual risk or that ECS does not guarantee future children will not have genetic conditions.^28^ A review study demonstrates that most carrier couples decide to change their reproductive plans to avoid conceiving a child with a severe genetic condition.^29^ Due to the (expected) absence of positive ECS, we could not study this, nor was that our aim.

### Strengths and limitations

Strengths of our study are that we studied the ECS offered in a primary care setting with trained nongenetics professionals who previously were not experienced in genetic counseling or provision of carrier screening. Moreover, the ECS was offered to the eligible general population, we included unscreened couples, and we distinguished between those who declined the offer before and after counseling. One possible limitation is that the T0 scores may not represent a “true” baseline measurement, as participants had already received and possibly read the study information. Since the anxiety and worry scores at T0 were associated with the course of anxiety and worry over time, it is important to note that the mean T0 scores were comparable to levels reported in similar studies.^23,30,31^ Secondly, we analyzed individuals, not couples. Admittedly, assuming that couple responses were independent from each other is debatable. Although views regarding ECS between couple members are largely comparable,^5^ this does not necessarily imply that couple members' anxiety, worry, or decisional conflict are also correlated. Finally, individuals who did not proceed with testing were less likely to fill out the last survey. While this may affect generalizability, numbers were small and response did not seem to be selective.

### Future perspectives and conclusion

Further qualitative research, e.g., in depth interviews, could complement this quantitative study to investigate the sources of increased decisional conflict and uncertainty, regret, and false reassurance. Our ECS was couple-based, which probably contributed to the favorable psychological outcomes. Future research should clarify if disclosure of individual carrier results leads to misunderstanding of individual health implications, negative feelings of being a carrier, stigmatization, and more anxiety and worry.^32,33^ In our view, large-scale implementation of ECS in a nongenetics professional setting or as a programmatic offer and the aim of ECS justify a couple-based approach. Large-scale implementation of ECS should include a follow-up to determine the impact on anxiety, worry, and reproductive decisions in identified carrier couples.^34^ Mackenzie’s mission is one of the initiatives that study couple-based ECS and the psychological impact in a nationwide population-based pilot.

Given the feasibility of the offer,^16^ the 15% uptake,^15^ and the favorable psychological outcomes, we conclude that large-scale implementation of the couple-based offer within an appropriate setting could well be responsible: while some individuals reported temporarily clinically relevant distress, overall, the psychological outcomes in eligible couples are acceptable.

## Supplementary information

Supplementary Appendix 1

Supplementary tableS1

Supplementary tableS2

Supplementary tableS3

Supplementary tableS4

Supplementary tableS5

Supplementary tableS6

Supplementary tableS7

## Data Availability

Relevant data included in this paper is available upon request.
