# Supplementary material for: Couple-based expanded carrier screening provided by general practitioners to couples in the Dutch general population: psychological outcomes and reproductive intentions
Source: Genet Med. 2021 Jun 10;23(9):1761–8. doi: 10.1038/s41436-021-01199-6 (PMC8460434; doi:10.1038/s41436-021-01199-6)
Supplement: Supplementary file 1 — Supplementary Appendix 1 [file 41436_2021_1199_MOESM1_ESM.docx]

**APPENDIX 1.** Psychological outcome measures and influencing factors

**Psychological outcome measures
*Anxiety***

Anxiety was measured using the 6-item short form State-Trait Anxiety Inventory (STAI-6). The response mode is a 4-point scale (1=not at all, 4=very much). The unweighted STAI-6 sum score range is 6-24. (1). A higher score indicates a higher level of anxiety. Scores on the STAI-6 were transferred to prorated 20-item STAI scores by multiplying by 20/6 (score range 20-80). The convergent validity of the Dutch form of the short STAI with the 20-item full STAI showed a correlation of 0.95 (2). The STAI-6 has been used previously to measure anxiety in general practice preconception care, demonstrating an average of 36.4 (95%CI of mean 35.4-37.3) for women aged 18-40 (3). A cut-off of 40 was used to indicate clinically relevant elevated levels of anxiety (4). Cronbach’s αs varied from 0.78 to 0.87 over the time points.

*Worry*

Given the lack of validated instruments to measure worry regarding being a carrier couple, we adapted the 6-item Cancer Worry Scale to this purpose (5). (see supplementary Table S2). Items were measured on a 4-point Likert scale (almost never-almost always), with scores ranging from 6 to 24. A higher score indicates a higher level of worry about being a carrier couple. Cronbach’s αs ranged from 0.69 (moderate/reasonable) to 0.84 (good) over the time points.

Decision-making

*Decisional conflict*

Decisional conflict regarding test participation was measured using the 16-item Decisional Conflict Scale (DCS) after counselling (T1), after testing (T2) and at T3, which was 6 months after receiving the test-offer (T0) for test-offer decliners; or at 6 months after T1 for the test-acceptors and test-decliners. DCS aims to measure decisional uncertainty, factors contributing to uncertainty and perceived effective decision-making (6,7). The response mode is a five-point scale (0= totally disagree, 4=totally agree). Cronbach’s αs varied between 0.84 and 0.94 over time. Individual scores were summed, divided by 16 and multiplied by 25 to obtain the total DCS score (score range: 0-100). A higher score indicates more decisional uncertainty/conflict. Reference values are available: no decisional conflict (scores below 25), moderate decisional conflict (scores between 25-≤ 37.5), and high degree of decisional conflict (scores above 37.5)(8).

*Anticipated regret and satisfaction with decision making*

Anticipated decisional regret at T0 was measured with one item : ‘I think that I would regret not having taken part in this test-offer later on’ (response mode, 5-point Likert scale: 1=totally disagree, 5=totally agree). A higher score indicates more anticipated regret. Scores were dichotomized into regret (scores ≥4) and neutral/no regret (scores ≤3). Whether the test result affected reproductive intentions was measured as no change, certainly change, possible change, unsure.
Being satisfied with one’s decision at T3 whether to undergo couple-based ECS was measured using the item “I am satisfied with my decision” from the DCS. Scores were dichotomized into dissatisfied (scores ≤2) and neutral/satisfied (scores ≥3).

Factors potentially influencing psychological outcomes

Factors hypothesised to potentially influence psychological outcomes were sociodemographic variables, perceived control over life, relationship variables, timing of next pregnancy, self-rated health, presence of a chronic condition in one of the parents, experiences with hereditary conditions, genetic counselling and testing, and intention to take part in the ECS test-offer (T0) (9,10).

*Sociodemographic variables* In survey T0, we recorded demographic characteristics regarding age, sex, relationship status, having children and educational level. Participants’ educational level and marital status were classified according to the Statistics Netherlands (CBS) definitions and summarized as in (11).

*Perceived control*Survey T0 also included the concept of mastery as a factor potentially influencing psychological outcomes. Perceived control is defined as someone’s perception of being in control over situations or circumstances in their lives. Perceived control was measured using the 7-item Pearlin Mastery Scale (PMS) (12). The response mode is a 5-point scale (1=totally agree, 5=totally disagree) with total PMS scores ranging from 7-35 after recoding. A higher score indicates a higher sense of self-mastery. Individuals with a lower PMS score are more prone to stress.

Reproductive intentions

Reproductive intentions were measured using the survey item reported previously by Lakeman et al., (2008)(13), which asked whether the test-result influenced participants’ reproductive plans or ideas about having children. If that was the case, participants were asked to indicate one reason out of six options to describe the change, e.g. ‘I am surer about having children’ or ‘I have more doubts about having a child’.

1. Marteau TM, Bekker H. The development of a six‐item short‐form of the state scale of the Spielberger State—Trait Anxiety Inventory (STAI). Br J Clin Psychol. 1992;31:301–6.

2. Van der Bij AK, De Weerd S, Cikot RJLM, Steegers EAP, Braspenning JCC. Validation of the Dutch Short Form of the State Scale of the Spielberger State-Trait Anxiety Inventory: Considerations for Usage in Screening Outcomes. Public Health Genomics. 2003;6:84–7.

3. De Jong-Potjer LC, Elsinga J, Le Cessie S, Van der Pal-De Bruin KM, Knuistingh Neven A, Buitendijk SE, et al. GP-initiated preconception counselling in a randomised controlled trial does not induce anxiety. BMC Fam Pract. 2006;7:66-76.

4. Annema C, Roodbol PF, Van den Heuvel ER, Metselaar HJ, Van Hoek B, Porte RJ, et al. Trajectories of anxiety and depression in liver transplant candidates during the waiting-list period. Br J Health Psychol. 2017;22:481–501.

5. Lerman C, Trock B, Rimer B, Boyce A, Jepson C, Engstrom P. Psychological and behavioral implications of abnormal mammograms. Ann Intern Med. 1991;114:657-61.

6. O’Connor AM. Validation of a Decisional Conflict Scale. Med Decis Mak. 1995;15:25–30.

7. Koedoot N, Molenaar S, Oosterveld P, Bakker P, De Graeff A, Nooy M, et al. The decisional conflict scale: further validation in two samples of Dutch oncology patients. Patient Educ Couns. 2001;45:187–93.

8. 1993 (updated 2010). Ottawa Hospital Research Institute. User Manual-Decisional Conflict Scale [Internet]. Available from: https://decisionaid.ohri.ca/docs/develop/User_Manuals/UM_Decisional_Conflict.pdf [accessed 04-10-2019]

9. Plantinga M, Birnie E, Abbott KM, Sinke RJ, Lucassen AM, Schuurmans J, et al. Population-based preconception carrier screening: how potential users from the general population view a test for 50 serious diseases. Eur J Hum Genet. 2016;24:1417–23.

10. Ioannou L, McClaren BJ, Massie J, Lewis S, Metcalfe SA, Forrest L, et al. Population-based carrier screening for cystic fibrosis: a systematic review of 23 years of research. Genet Med. 2014;16:207–16.

11. Schuurmans J, Birnie E, Ranchor AV, Abbott KM, Fenwick A, Lucassen A, et al. GP-provided couple-based expanded preconception carrier screening in the Dutch general population: who accepts the test-offer and why? Eur J Hum Genet. 2020;28:182–92.

12. Pearlin LI, Schooler C. The structure of coping. J Health Soc Behav. 1978;19:2–21.

13. Lakeman P, Plass AM, Henneman L, Bezemer PD, Cornel MC, Ten Kate LP. Three-month follow-up of Western and non-Western participants in a study on preconceptional ancestry-based carrier couple screening for cystic fibrosis and hemoglobinopathies in the Netherlands. Genet Med. 2008;10:820–30.
