# Supplementary material for: Couple-based expanded carrier screening provided by general practitioners to couples in the Dutch general population: psychological outcomes and reproductive intentions
Source: Genet Med. 2021 Jun 10;23(9):1761–8. doi: 10.1038/s41436-021-01199-6 (PMC8460434; doi:10.1038/s41436-021-01199-6)
Supplement: Supplementary file 2 — Supplementary tableS1 [file 41436_2021_1199_MOESM2_ESM.docx]

**Table S1. Overview of concepts and measurements**

| Concepts/instruments | T0 after receiving the offer for counselling and testing | T1 after GP counselling/ before testing | T2 after testing | T3  six months after T0 or T1 |
| --- | --- | --- | --- | --- |
| Socio demographic variables | X |  |  |  |
| Self-rated health/experiences of genetic chronic conditions, genetic conditions and genetic testing | X |  |  |  |
| Mastery  Pearlin Mastery Scale (PMS) | X |  |  |  |
| Anxiety  6-item State-Trait Anxiety Index (STAI) | X | X | X | X |
| Worry   Adapted 6-item Cancer Worry Scale | X | X | X | X |
| Decisional conflict  Decisional Conflict Scale (DCS)  Satisfaction with decision  Anticipated regret | X | X | X | X  X |
