# Supplementary material for: Couple-based expanded carrier screening provided by general practitioners to couples in the Dutch general population: psychological outcomes and reproductive intentions
Source: Genet Med. 2021 Jun 10;23(9):1761–8. doi: 10.1038/s41436-021-01199-6 (PMC8460434; doi:10.1038/s41436-021-01199-6)
Supplement: Supplementary file 3 — Supplementary tableS2 [file 41436_2021_1199_MOESM3_ESM.docx]

**Table S2. STAI, worry and reproductive intentions

6-item STAI**A number of statements which people have used to describe themselves are given on the

following pages. Read each statement and then select the appropriate button to indicate

how you feel ***right*** now, that is, ***at this moment***. There are no right or wrong answers. Do not

spend too much time on any one statement but give the answer which seems to describe

your present feelings best.
(Response mode: not at all / somewhat/ moderately so / very much so)

- Calm
- Tense
- Upset
- Relaxed
- Content
- Worried

**Adapted 6-item worry scale**

- How often have you thought about the chances of being a carrier couple?
- How often have your thoughts about the chances of being a carrier couple influenced your mood?
- How often have your thoughts about the chances of being a carrier couple restricted you in your daily activities?
- How worried are you about the possibility of being a carrier couple?
- How often have you been worried about the chances of being a carrier couple?
- To what extent is this worry a problem for you?

**Reproductive intentions**

1: ‘Did the test-results change your ideas about having children?’
(Response mode: Yes / No / Possibly / I do not know)

2: If yes or possibly, participants were asked to tick one option out of the following:

- I am surer about having children,
- I now have doubts about having a child,
- I want more children than I did before the carrier testing,
- I want less children than I did before the carrier testing,
- I now definitely do not want any (more) children,
- My ideas have changed in other ways
