# Supplementary material for: Couple-based expanded carrier screening provided by general practitioners to couples in the Dutch general population: psychological outcomes and reproductive intentions
Source: Genet Med. 2021 Jun 10;23(9):1761–8. doi: 10.1038/s41436-021-01199-6 (PMC8460434; doi:10.1038/s41436-021-01199-6)
Supplement: Supplementary file 4 — Supplementary tableS3 [file 41436_2021_1199_MOESM4_ESM.docx]

**Table S3. STAI and Worry scores at T0**

|  | **Group 1 test-offer decliners  n=120** | **Group 2 test-decliners    n=26** | **Group 3 test-acceptors  n=234** | **Group 1 vs Groups 2+3** | **Groups 1+2 vs Group 3** |
| --- | --- | --- | --- | --- | --- |
| **STAI***^a^* |  |  |  |  |  |
| Mean (SD)*^b^* | 33.68 (10.12) | 32.64  (11.42) | 30.74  (8.17) | 33.68 (10.12) vs.  30.92 (8.51) | 33.47 (10.35) vs.  30.74 (8.17) |
| Comparison of subgroups |  |  |  | 2.76  (95%CI 0.65-4.87)  P=.010 | 2.73  (95%CI: 0.75-4.71)  p=.007 |
| Cohen’s d |  |  |  | 0.32 | 0.19 |
| STAI ≥40 | 26 (27.1%) | 6 (25.0%) | 35 (15.0%) | 27.1% vs. 15.9%,  p=.017 | 26.7% vs. 15.0%,  p=.008 |
| **Worry***^c^* |  |  |  |  |  |
| Median (IQR) | 6.0 (6.0-7.0) | 6.0 (6.0-8.5) | 6.0 (6.0-7.0) |  |  |
| Comparison of subgroups |  |  |  | 6.0 (6.0-7.0)  vs.  6.0 (6.0-7.0),   p>.99 | 6.0 (6.0-7.0)  vs. 6.0 (6.0-7.0),  p=.80 |
| Cohen’s d |  |  |  | 0.03 | 0.35 |

STAI, State-Trait Anxiety Inventory; SD, standard deviation; IQR, interquartile range;
*^a^*Missing data: 24 individuals (test-offer decliners) and 2 individuals (test-decliners)
*^b^*Significantly lower than the reference value of mean 36.4 (95%CI of mean: 35.4-37.3) for the test-offer decliners (p=.02) and test-acceptors (p<.001); comparable to the reference value for the test-decliners (p=.09)
*^c^*Missing data: 25 individuals (test-offer decliners) and 2 individuals (test-decliners)
