# Supplementary material for: Couple-based expanded carrier screening provided by general practitioners to couples in the Dutch general population: psychological outcomes and reproductive intentions
Source: Genet Med. 2021 Jun 10;23(9):1761–8. doi: 10.1038/s41436-021-01199-6 (PMC8460434; doi:10.1038/s41436-021-01199-6)
Supplement: Supplementary file 5 — Supplementary tableS4 [file 41436_2021_1199_MOESM5_ESM.docx]

**Table S4. STAI and Worry scores at T3**

|  | **Group 1 test-offer decliners  n=120** | **Group 2 test-decliners**    **n=26** | **Group 3 test-acceptors   n=234** | **Group 1 vs group 3** | **Group 2 vs group 3** | **Groups 1+2 vs group 3** | **Group 1 vs groups 2+3** |
| --- | --- | --- | --- | --- | --- | --- | --- |
| **STAI***^a^* |  |  |  |  |  |  |  |
| Mean (SD), Mean difference (95%CI of mean difference) | 35.93***^b^*** (10.70) | 34.07*^b^* (15.26) | 28.84 *^b^*  (8.43) | 7.08  (4.13-10.03) | 5.23 (-6.53- 16.99) | 6.77  (3.42-10.13) | 6.82  (3.78-9.87) |
| p-value*^c^* |  |  |  | p<.001 | P=.34 | P<.001 | P<.001 |
| Cohen’s d |  |  |  | 0.79 | 0.59 | 0.74 | 0.74 |
| STAI ≥40 | 17 (37.8%) | 2  (22.2%) | 22 (12.7%) | 37.8%  vs.  12.7% | 22.2%  vs  12.7% | 35.2%  vs.  12.7% | 37.8% vs. 13.2% |
| P-value*^d^* |  |  |  | P<.001 | P=.61 | P<.001 | P<.001 |
| **Worry***^e^* |  |  |  |  |  |  |  |
| Median (IQR) | 6 (6-7) | 6 (6-7.5) | 6 (6-7) |  |  | 6 (6-7)  vs  6 (6-7) | 6 (6-7) vs  6 (6-7) |
| P-value*^f^* |  |  |  | P=.59 | P=.61 | P=.50 | P=.63 |
| Cohen’s d |  |  |  | 0.02 | 0.18 | 0.05 | 0.02 |

STAI, State-Trait Anxiety Inventory; SD, standard deviation; IQR, interquartile range;
*^a^* Missing data: 75 individuals (test-offer decliners), 17 individuals (test-decliners) and 61 individuals (test-acceptors)
*^b^*Comparison to the reference value of mean 36.4 (95%CI of mean: 35.4-37.3)(19) for test-offer decliners (p=.77) and test-decliners (p=.51); significantly lower than the reference value for test-acceptors (p<.001)
*^c^*Differences tested with independent Student’s t-test
*^d^*Differences tested with Fisher’s Exact test (test-decliners vs test-acceptors) or Pearson’s chi-square test (other comparisons)
*^e^*Missing data: 76 individuals (test-offer decliners), 17 individuals (test-decliners) and 61 individuals (test-acceptors).
*^f^*Differences between groups tested with the non-parametric Mann-Whitney U test
