# Supplementary material for: Couple-based expanded carrier screening provided by general practitioners to couples in the Dutch general population: psychological outcomes and reproductive intentions
Source: Genet Med. 2021 Jun 10;23(9):1761–8. doi: 10.1038/s41436-021-01199-6 (PMC8460434; doi:10.1038/s41436-021-01199-6)
Supplement: Supplementary file 6 — Supplementary tableS5 [file 41436_2021_1199_MOESM6_ESM.docx]

**Table S5. Change of STAI and Worry scores between T0 and T3**

|  | **Test-offer decliners  n=120** | **Test-decliners**  **n=26** | **Test-acceptors  n=234** |
| --- | --- | --- | --- |
| **STAI***^a^* |  |  |  |
| Mean within group difference (95%CI)*^b,c^* | 1.04  (95%CI: -1.79 - 3.86) | -3.33  (IQR: -6.67 -15.00) | 0.0  (IQR: -6.67 - 3.33) |
| p-value*^c^* | P=0.46 | P=0.87 | P=0.015 |
| Cohen’s d | 0.1 | 0.3 | 0.2 |
| **Worry***^a^* |  |  |  |
| Median within group difference (IQR)*^b^* | 0 (-1 - 0) | 0 (-5 - 0) | 0 (0 - 1) |
| P-value*^d^* | P=.33 | P=.14 | P=.90 |
| Cohens’ d | 0.20 | 0.42 | 0.03 |

STAI, State-Trait Anxiety Inventory; SD, standard deviation; IQR, interquartile range;
*^a^*Missing data: 76 individuals (test-offer decliners), 17 individuals (test-decliners) and 61 individuals (test-acceptors)
*^b^*Differences defined as T3 score minus T0 score. A positive difference indicates that the mean STAI score at T3 is higher than the mean STAI at T0.
*^c^*Data for test-offer decliners presented as mean difference (95%CI of mean difference), for the test-decliners and test-acceptors as median difference (IQR of difference). Within group difference tested with the paired Student’s t-test (test-offer decliners) or the Wilcoxon matched pairs test (the test-decliners and test-acceptors)
*^d^*Within group difference in rank orderings tested with the Wilcoxon matched pairs test.
