# Supplementary material for: Couple-based expanded carrier screening provided by general practitioners to couples in the Dutch general population: psychological outcomes and reproductive intentions
Source: Genet Med. 2021 Jun 10;23(9):1761–8. doi: 10.1038/s41436-021-01199-6 (PMC8460434; doi:10.1038/s41436-021-01199-6)
Supplement: Supplementary file 7 — Supplementary tableS6 [file 41436_2021_1199_MOESM7_ESM.docx]

**Table S6. Repeated Measurements Analysis of STAI and Worry scores by group over time**

| **Variable** | **STAI score  beta (95%CI)***^a^* | **p- value** | **transformed Worry score***^b^* **beta (95%CI)***^a^* | **p- value** |
| --- | --- | --- | --- | --- |
| Time-effect:   T1: after GP counselling before testing  T2: after testing  T3: 6 months after T0/T1 (reference) | .33 (-.94 ; 1.61) .16 (-1.14 ; 1.45) 0 | .61 .81 – | -.03 (-.12 ; .06) -.09 (-.18 ; .01) 0 | .50 .068 – |
| Group-effect:  G1: Test-offer decliners  G2: Test-decliners   G3: Test acceptors (reference) | 4.23 (1.27 ; 7.19) 3.52 (-2.04 ; 9.09) 0 | .005 .22 – | -.13 (-.31 ; .06) -.52 (-.87 ; -.18) 0 | .18 .003 – |
| STAI baseline score | .49 (.39 ; .59) | <.001 | – | – |
| Transformed worry baseline score | – | – | .15 (.12 ; .18) | .001 |
| Male (reference: female) | -.34 (-1.80 ; 1.12) | .65 | -.09 (-.19 ; .00) | .056 |
| Interaction-effect Time x Group:   T1 x test-decliners | -.44 (-6.34 ; 5.46) | .88 | .63 (.21 ; 1.05) | .003 |

STAI, State-Trait Anxiety Inventory; CI, confidence interval of beta-coefficient; AIC, Akaike’s Information Criterion
*^a^*Independent variables were time, group, STAI/worry score at T0, sex and interaction effect time x group. Adjustment covariables were educational level, self-rated health status, intention to accept the offer, marital status, timing of next pregnancy and the Pearlin Mastery Scale (PMS) baseline score.
*^b^*Transformation: transformed Worry score = natural Log(-5+ raw Worry score), score range: 0 to 2.94; a higher transformed worry score indicates more worry
Goodness of fit: AIC is 3950.91 (STAI score), and 852.39 (transformed Worry score)
