# Supplementary material for: Couple-based expanded carrier screening provided by general practitioners to couples in the Dutch general population: psychological outcomes and reproductive intentions
Source: Genet Med. 2021 Jun 10;23(9):1761–8. doi: 10.1038/s41436-021-01199-6 (PMC8460434; doi:10.1038/s41436-021-01199-6)
Supplement: Supplementary file 8 — Supplementary tableS7 [file 41436_2021_1199_MOESM8_ESM.docx]

**Table S7. Decisional conflict and satisfaction with decision-making**

|  | **Test-offer decliners n= 120** | **Test-decliners**  **n=26** | **Test-acceptors**  **n=234** | **Total**  **n=380** |
| --- | --- | --- | --- | --- |
| **Decisional conflict***^a,b^* T1: Low  Moderate  High  T2: Low  Moderate  High  T3: Low*^c^*   Moderate  High | NA NA NA  NA NA NA   9 (20.5%) 18 (40.9%) 17 (38.6%) | 7 (70.0%) 3 (30.0%)  0 ( 0.0%)  NA NA NA  4 (44.4%) 4 (44.4%) 1 (11.1%) | 116 (50.9%)  95 (41.7%)  17 ( 7.5%)  139 (72.8%)  49 (25.7%)  3 ( 1.6%)  94 (55.0%) 62 (36.3%) 15 ( 8.8%) | 123 (51.7%)  98 (41.2%)  17 ( 7.1%)  139 (72.8%)  49 (25.7%)  3 ( 1.6%)  107 (47.8%)  84 (37.5%)  33 (14.7%) |
| **Satisfaction with  decision making***^d^* |  |  |  |  |
| T0: Anticipated regret*^e^*  T3: Dissatisfied with decision  Satisfied with decision | N=91 33 (36.3%)  4 ( 8.9%)  41 (91.9%) | N=24  14 (58.3%)  0 ( 0.0%)  9 (100.0%) | N=232  127 (54.7%)  1 ( 0.6%)  172 (99.4%) | N=347  174 (50.1%)  5 ( 2.2%)  222 (97.8%) |
|  |  |  |  |  |

NA, not applicable *^a^*Missing data test-offer decliners: 76 individuals at T3; test-decliners: 16 individuals at T1, 17 at T3; test-acceptors: 6 individuals at T3, 43 at T2, 63 at T3.
*^b^*Decisional conflict score defined as moderate: 25-37.5, and high: >37.5 (22)
*^c^*Test-offer decliners vs. test-acceptors, p<.001 (Pearson Chi-square); test-decliners vs. test-acceptors, p=.67 (Fisher’s exact test)
*^d^*Missing data at T0: 39 (test-offer decliners), 2 (test-decliners), 2 (test-acceptors) individuals. Missing data at T3: 75 (test-offer decliners), 17 (test-decliners), 61 (test-acceptors) individuals.
*^e^*Test-offer decliners vs. test-acceptors, p<.001 (Pearson Chi-square); test-decliners vs. test-acceptors, p=.87 (Pearson Chi-square); test-offer decliners vs. test-decliners , p=.04 (Pearson Chi-square)
